# The Biodegradation Role of *Saccharomyces cerevisiae* against Harmful Effects of Mycotoxin Contaminated Diets on Broiler Performance, Immunity Status, and Carcass characteristics

**DOI:** 10.3390/ani10020238

**Published:** 2020-02-03

**Authors:** Muhammad Arif, Atia Iram, Muhammad A. K. Bhutta, Mohammed A. E. Naiel, Mohamed E. Abd El-Hack, Sarah I. Othman, Ahmed A. Allam, Mahmoud S. Amer, Ayman E. Taha

**Affiliations:** 1 Department of Animal Sciences, College of Agriculture, University of Sargodha, Punjab 40100, Pakistan; arif.inayat@uos.edu.pk (M.A.); dr_sadan@yahoo.com (A.I.); aryanmuhammad08@gmail.com (M.A.K.B.); 2 Department of Animal Production, Faculty of Agriculture, Zagazig University, Zagazig 44511, Egypt; 3 Poultry Department, Faculty of Agriculture, Zagazig University, Zagazig 44511, Egypt; 4 Biology Department, College of Science, Princess Nourah bint Abdulrahman University, P.O. Box 24428, Riyadh 11671, Saudi Arabia; sialothman@pnu.edu.sa; 5 Zoology Department, Faculty of Science, Beni-Suef University, Beni-Suef 65211, Egypt; allam1081981@yahoo.com; 6 Laser Application in Biotechnology Department, National Institute of Laser Enhanced Science, Cairo University, Cairo 12613, Egypt; lasthero_2020@yahoo.com; 7 Department of Animal Husbandry and Animal Wealth Development, Faculty of Veterinary Medicine, Alexandria University, Edfina 22758, Egypt; Ayman.Taha@alexu.edu.eg

**Keywords:** biodegradation, *Saccharomyces cerevisiae*, performance, mycotoxin

## Abstract

**Simple Summary:**

Over the past two decades, the use of agents for the biodegradation of mycotoxins has led to a reduction in their accumulation and toxicity in the digestive tract of animals. Thus, mycotoxin decontaminating agents are very useful in the prevention of aflatoxicosis. The present feeding trial aimed to evaluate the biodegradation role of *Saccharomyces cerevisiae* in the prevention of the harmful effects of a mycotoxin contaminated diet on broiler performance, immunity, and carcass traits. The obtained results revealed significant improvements in broiler growth performance parameters, carcass traits, and antibody titer against infected diseases as an effect of the dietary inclusion of *Saccharomyces cerevisiae* up to 3.75 g kg^−1^. Consequentially, it could be used in broiler contaminated diets without negatively affecting bird health.

**Abstract:**

A feeding trial (35 days) was carried out to investigate the effect of *Saccharomyces cerevisiae* cell wall as a mycotoxin biodegradation agent on the performance, feed efficiency, carcass traits, and immunity response against diseases in broilers fed aflatoxin B1 contaminated diets. For this purpose, 200 one day old broilers were randomly allotted into four groups, each with five replicates (10 birds per replicate). Four starter and finisher experimental rations were formulated by using (A) 0, (B) 1.25, (C) 2.5, and (D) 3.75 g kg^−1^ of *Saccharomyces cerevisiae*. Experimental diets were contaminated with aflatoxin B1 (100 ppb kg^−1^ diet). The experimental chicks were kept under standard managerial conditions, and the vaccination program was followed against infectious bursal disease (IBD), infectious bronchitis (IB), and Newcastle disease (ND) diseases. At the end of the feeding trial, carcass, organ weight, and blood samples were collected randomly to determine the carcass traits and antibody titer against ND and IBD viruses. Throughout the experiment, the addition of 3.75 g kg^−1^ of the *Saccharomyces cerevisiae* cell wall (Group-D) in feed resulted in the highest weight gain, final weight, feed intake, and the lowest FCR values followed by C group compared with the other groups. All carcass traits were significantly (*p* > 0.05) improved by increasing the inclusion levels of *Saccharomyces cerevisiae* in broiler diets. It could be concluded that the broiler diet supplemented with 2.5 or 3.75 g kg^−1^ of *Saccharomyces cerevisiae* as a biodegrading agent resulted in improved growth performance, immunity activity and carcass traits, and supplementation with *Saccharomyces cerevisiae* at these levels can be used effectively in broiler diets without negatively affecting bird health status.

## 1. Introduction

Globally, the poultry industry is one of the major agricultural subsectors [1]. According to an Food and Agriculture Organization of the United Nations (FAO) report, there is a total of about 1210 million, including 87.16, 1057.65, 52.2, and 12.39 million rural, meat type, egg type, and breeding stock of poultry birds, respectively [2]. In developed countries, poultry breeders play an important role in the conservation of the genetic diversity of poultry species (meat-type birds), but also contribute in the production of adequate quantities of high-quality animal protein that are necessary for covering the consumption needs of the rapidly increasing human population [3]. In Pakistan, meat from poultry is about 32.7% of the total meat available in the country for human consumption [4]. Due to the existence of anti-nutritional agents and toxins in diets, the poultry industry faces many problems, such as suppressed growth rates, feed efficiency, and higher mortality rates [5]. Fungus spoilage is the main factor that deteriorates the hygiene of poultry feed [6]. Bad harvesting methods, higher ambient temperatures, and inadequate drying of cereals in the field provide favorable conditions for the growth of fungus [7]. In addition, the seasonal supply of cereals results in the storage of grains for longer periods, which worsens the problem [8]. This leads to increased development of secondary fungal metabolites associated with mycotoxin production [9].

Mycotoxin contamination is widespread in plant products, particularly in grains, fruits, hazelnuts, almonds, seeds, animal feed, and other agro-industrial products or foods intended for animal or human consumption [10]. Currently, among the 300 known mycotoxins, only aflatoxin, zearalenone, deoxynivalenol, fumonisin, and botulinum are at the forefront of research interest due to serious public health problems and their widespread existence in feed ingredients [11]. Mycotoxins pose a huge risk to livestock health as well as human health due to their numerous toxic effects and their possible synergistic properties [12]. When animals consume food contaminated with high concentrations of mycotoxins, often reduced animal productivity (reduced body weight gain, reduced litter sizes, deformed offspring, reduced egg production), and immune suppression is observed with severe economic losses [13]. The secondary metabolites of *Aspergillus flavus*, *Aspergillus parasiticus*, *Aspergillus ochraceus*, and *Fusarium sporotrichoides* are Aflatoxin (AF), Ochratoxin A (OA), and T-2 toxin (T-2) [14]. Aflatoxins, a class of mycotoxins produced by fungal species, are often found in feed ingredients used for poultry rations [15]. Aflatoxin affects all types of poultry species, and the mortality rate is relatively high since even low levels can be dangerous if they are consistently fed over a period of time, particularly in chicken and turkeys [15]. Hussain et al. [16] suggested that growing poultry should not ingest daily aflatoxin levels above 20 mg through the diet. Nevertheless, daily intake of less than 20 mg of aflatoxin has been reported to reduce disease resistance, disrupt stress regulation, and induce bruising in broilers [17]. If the birds are exposed to aflatoxin for longer periods, toxicity causes further damage to the hepatocytes, tissue, and intestinal tract [18].

Many treatments are recommended for the prevention of aflatoxicosis, i.e., biological, chemical, and physical methods that can be separated into pre- and post-harvest technologies [19]. Mycotoxin detoxification by microorganisms has gained interest as a biological method and has been well studied by researchers [20]. Several studies found that AFB1 binding to the bacterial cell wall is strain-specific [21]. Yeasts occur as part of the natural microbial population in spontaneous food fermentation and as starting crops in the beverage industries [22]. One of the most important microorganisms in food fermentation, *Saccharomyces cerevisiae*, has been shown to strongly bind specific mycotoxins to cell wall components [23,24]. In addition to its excellent nutritional value, the yeast or yeast cell walls have the ability to bind mycotoxins [25]. The adsorption of mycotoxins can be increased by using only yeast cell walls instead of whole cells [26]. The cell walls with polysaccharides (glucan, mannan), proteins, and lipids show numerous different and easily accessible adsorption sites as well as numerous binding mechanisms, such as hydrogen bonds, ionic, or hydrophobic interactions [27]. Recently, it has been shown that the 10 β-d-glucan fractions of the yeast cell wall are actively involved in the zearalenone binding process and that the β-d-glucan structurally regulates the binding capacity [28]. The components of carbohydrates tend to be unique binding sites, with several toxins having various binding sites [29].

These substances (toxin-binder) are non-digestible and are claimed to be effective in binding with mycotoxins in the small intestines when mixed with broiler feed [30]. Thus, the current trial was designed to investigate the biodegradable effects of mycotoxin binding agents (*Saccharomyces cerevisiae* cell wall) on the performance, immune status, and carcass traits of broilers fed mycotoxin contaminated diet in starter and finisher feeding period.

## 2. Materials and Methods

The experimental animal procedure was accompanied by recommendations for animal use and care from the National Institute of Health (NIH) and endorsed by the local ethical committee of the University of Sargodha, Pakistan.

### 2.1. Experimental Design and Management 

A total of two hundred healthy male day-old ROSS-308 broiler chicks were obtained from a private hatchery, individually weighed (initial weight), and then allocated into four groups under a full complete randomized design. Each group consisted of 5 replicates with 10 birds. The trial’s overall duration was 35 days. The experiment was implemented at the broiler farm belonging to the Department of Animal Sciences, College of Agriculture, University of Sargodha. Regularly, dust in the experimental shed was removed by an air-cleaner and water. The shed was allowed to air-dry, and then phenol (Prophyl 75^®^, LAB. OVEJERO SA, Spain) was applied. Finally, the facility was disinfected by pump spraying with a combination of formalin and water at the ratio of 1:10. Before the chicks arrived, fumigation was also done.

Using the KMnO_4_ solution, feeders and drinkers were washed and dried in sunlight to ensure maximum decontamination. Chicks were placed into individual pens with the same conditions of rearing management in each replication. In the floor littered system, rice husks were used as bedding materials.

Measures for biosecurity were strictly followed, and a trial was implemented under fully hygienic conditions. Drinkers were regularly cleaned and washed. The lighting program followed standard ROSS TECH Lighting for Broilers [31]. Shed temperature was set for 1 week to 95 °F, and then every week, it was decreased by 5 °F until it reached 75 °F. The all rearing condition was under control during the feeding trial period.

### 2.2. Toxin Binder Structure 

The toxin binder used in this trial was LANSAIL (the cell wall of *Saccharomyces cerevisiae*). Each one kilogram from the product contains 80% yeast cell wall, 10% β glucan, and 10% mannooligosaccharides. Biological toxin binder (LANSAIL) produced by Shandong Bio Sunkeen Co., LTD.

### 2.3. Mycotoxin Preparation Method 

*Aspergillus flavus* MD 341 was obtained from the Department of Plant Pathology and Soil, College of Agriculture, University of Sargodha. For 8 days, the above-mentioned fungus was incubated on liquid media containing 2% yeast extract and 20% sucrose for aflatoxin B1 production. The resulted aflatoxin B1 concentration in media was determined using the method of The Official Methods of Analysis (A.O.A.C.) [32]. The required levels of aflatoxin B1 in the diets were obtained by spraying with a liquid media that contained this mycotoxin at the selected levels.

### 2.4. Diet Preparation and Feeding Regime 

Two formulated and balanced rations (starter and finisher) that met the broiler chicken requirements, according to the National Research Council [33], were used as presented in Table 1. All treated broiler (one-day-old) were fed aflatoxin B1 (100 ppb kg^−1^ diet) contaminated diet until the end of the feeding trial. Broiler chickens were randomly allocated into four experimental groups as follows: A, fed contaminated AFB1 diet; B, C, and D fed contaminated diet further supplemented with 1.25, 2.5, or 3.75 g kg^−1^ of LANSAIL (the cell wall of *Saccharomyces cerevisiae*) as a biodegrading agent, respectively. All the birds had ad libitum access to feed and water. 

The determination of crude protein in the diet was performed using the Kjeldahl method, as described by A.O.A.C. [32]. The diet metabolizable energy values were corrected by nitrogen balance and calculated based on Sakomura and Rostagno [34] study. In addition, digestible lysine was calculated according to the following formula: Digested lysine = (feed intake × %lysine in the diet) − fecal lysine [35]. 

### 2.5. Health and Vaccination Programs 

Biosecurity measures, including foot dipping by disinfectants and cleaning of the shed, were applied after the arrival of chicks until their slaughter. Only farm personnel were allowed to enter the experimental facility by following biosecurity protocols every day. Strict hygiene and biosecurity measures were adopted during the life span of broilers. Birds were vaccinated against Newcastle disease, avian influenza virus H9, infectious bronchitis, and infectious bursal disease, as shown in Table 2.

The shed was divided into twenty pens. Dimensions of each pen were 5 × 3 × 2.5 feet. Rice husk was used as a bedding material in each pen to keep the floor sufficiently dry.

### 2.6. Performance and Mortality Rate 

Day-old chicks’ live body weights were recorded in all replicates individually and at the end of each week. Weights were, therefore, used to calculate the mean body weight. Feed intake was assessed on a weekly basis as follows; Feed intake = (Feed offered − Feed refused)/Number of birds per replicate. Feed conversion ratio (FCR) was calculated as feed intake (g)/weight gain (g). Mortality was also recorded throughout the trial period.

### 2.7. Carcass Measurements 

At the age of the 35th day, two birds were randomly selected from each replicate (10 birds per treated group) for carcass and visceral organ evaluation. Before slaughter, the feed was withdrawn for six hours to ensure that the digestive tract was empty, and the live body weight was determined. After slaughtering, the birds were defeathered and eviscerated. The carcass weight and the weight of the breast muscle and legs with paws were determined. The weight of the liver, heart, and gizzard was also recorded after removing skin, fat, and connective tissue. In addition, the intestine was emptied of its content before the measurement of its weight. In addition, carcass yield was calculated (carcass weight/live weight of bird) × 100, as well as the relative giblet weight by following: relative organ weight = (weight of organ/live weight of bird) × 100.

### 2.8. Serum Immune Response 

Blood samples were collected from 10 birds per group (2 birds per replicate) on 35th day from wing vein in a gel tube (SPS tubes/“yellow tops”) that contained a special type of gel that separates blood cells from serum and causes the blood to clot quickly. Samples were centrifuged for 15 min at 3000 rpm. For biochemical analysis, including titer against Newcastle disease (ND) and infectious bursal disease (IBD), these serum collection tubes were kept in deep-frozen storage. For test serum metabolites, the commercially available MicroLab300 Merck Analyzer (ELI Tech Group, France) was used.

### 2.9. Statistical Analysis 

Collected data were analyzed using SPSS (v16) software statistical analysis program [36]. The analyses of variances (one-way ANOVA) were performed among the experimental groups. Treatment means were compared by the least significance difference test. The orthogonal polynomial contrast was adopted to check the linear and quadratic effects of dietary levels of *Saccharomyces cerevisiae* [37]. 

## 3. Results

### 3.1. Performance and Mortality Rate

Values for growth performance, feed efficiency, and mortality rate are shown in Table 3. Body weight gain (BWG) and feed intake (FI) were linearly increased (*p* < 0.05). On the other hand, FCR was linearly reduced as an effect of the increasing levels of *Saccharomyces cerevisiae* in the diet. A quadratic increase was also shown for feed intake. The mortality was not significantly affected by toxin binder supplementation in the diet. However, in the group with the highest concentration of *Saccharomyces cerevisiae* in the diet, no mortality was recorded during the feeding period (D group).

### 3.2. Carcass Traits

The effects of dietary fortification with different levels of *Saccharomyces cerevisiae* as a biodegradation agent against mycotoxin contaminated diet on broiler carcass characteristics are presented in Table 4. The mean weights of live body, carcass, and heart were linearly increased (*p* < 0.01) with an increased level of *Saccharomyces cerevisiae* in the supplemented diet. The mean values of dressing percentage, breast muscle, and drumsticks weight were linearly and quadratically increased (*p* < 0.05) as the supplementation levels of *Saccharomyces cerevisiae* enhanced. The dietary inclusion of *Saccharomyces cerevisiae* did not induce any significant change in gizzard, proventriculus, and kidney relative weight. On the other hand, the liver and intestine weight were linearly and quadratically reduced by increasing the *Saccharomyces cerevisiae* level in the diet.

### 3.3. Immune Response against Infected Diseases

The mean titer of antibodies against ND and IBD infected diseases in different groups fed with mycotoxin contaminated diet and treated with several levels of *Saccharomyces cerevisiae* as a biodegrading agent are shown in Table 5. A linear effect was observed; broilers fed the diet with the highest level of *Saccharomyces cerevisiae* indicated higher antibody titers against ND and IBD infection diseases. 

## 4. Discussion

Mycotoxins cause a variety of diseases, commonly referred to as “mycotoxicoses,” either directly or in combination with other primary stressors, such as pathogens [38]. Acute cases induced by intestinal absorption of high mycotoxin levels may lead to high mortality and a marked decline in poultry growth represented by apparent clinical signs and post-mortem lesions [15]. In most cases, mycotoxicosis is caused by lower fungal metabolite ingestion, leading to a significant decline in performance and immunosuppression in broiler [39]. This feeding trial was designed to examine the effect of *Saccharomyces cerevisiae* supplemented diet as mycotoxin biodegradation agent on the growth performance, feed efficiency, carcass traits, and immunity response against diseases of broilers fed aflatoxin B1 contaminated diets.

Several studies demonstrated the harmful effects of aflatoxin-contaminated diets on broiler performance. Yarru et al. [40] and Sridhar et al. [41] found that broiler chicks fed diets contaminated with 1.0 mg of AFB1 per kg of diet showed significantly reduced feed intake and BW gain and increased relative liver weight. In addition, Valdivia et al. [42] demonstrated that contaminated broiler chicks (one-day-old) diets’ up to 3 mg AFB1 kg^−1^ diet for 21 days reduced feed intake and increased FCR values. The obtained results from our study coincide with the finding of Yildiz et al. [43], who stated that the addition of *Saccharomyces cerevisiae* (2 g kg^−1^ diet) to an aflatoxin (AF) contaminated diet significantly reduced the deleterious effects of AF on body weight gain, feed intake, egg production, egg weight, and feed conversion ratio in quail. In addition, Santin et al. [44] and Santin et al. [45] proved that the dietary supplementation with the cell wall of *Saccharomyces cerevisiae* improved the feed conversion of birds exposed or not to a low level of aflatoxin at 42 days of age. Beneficial effects of a biodegradable agent can result from its activity as a biodegradable agent with toxins that can act as an antioxidant by activating the production of enzymes and increasing the weight gain by increasing vitamin and mineral absorption and protein metabolization [46]. Additionally, biodegrading agent supplementation has an impact on the digestive tract, enhancing the development of digestive enzymes that play a vital role in improving digestion and, consequently, weight gain [47]. Likewise, biodegradation agent supplementation increases the length of the villi, reduces intestinal pH, reduces intestinal microbes, increases the secretion of auxiliary digestive enzymes, and improves nutrient absorption, leading to enhanced growth [48]. Moreover, Wu et al. [10] reported that *Saccharomyces cerevisiae* has the ability to bind to AF because of the presence of oligomannanes in the structure of the yeast cell wall. The oligomannanes’ chemical structure was able to bind 95% of AFB1. At the same time, Bueno et al. [49] suggested that the improved binding capability of *S. cerevisiae* towards AF may be due to the increased availability of binding sites. It is understood that toxin removal occurs through adhesion to components of the cell wall, rather than covalent binding or metabolic degradation, although the dead cells are still binding to toxin [23].

Ochratoxin and aflatoxin have been documented to lead to an increase in relative liver and kidney relative weight in poultry [50]. Our study results indicated significant improvement in the dressing percentage, live body, carcass, heart, and drumsticks, and breast muscle mean weight with the highest inclusion level of the biodegradation agent in the supplemented diet. Furthermore, there were no significant effects of *Saccharomyces cerevisiae* on gizzard, proventriculus, and kidney relative weight. These findings were in accordance with that of Singh et al. [51] who reported that dietary supplementation of an ochratoxin (150 ppb) contaminated feed with *S. Cerevisiae* (0.1 or 0.075 %) in combination with Vit E (100 or 200 mg kg^−1^) reduced the adverse effects of ochratoxicosis on carcass traits and organ weights (liver and thymus weights) of broiler chickens. Moreover, Kidd et al. [52] reported that when broiler breeders were fed *S. Cerevisiae* based products, the obtained progeny had a higher breast yield than the control group. The increase in cutting weight may be correlated with the use of *S. Cerevisiae* as a nutritional agent that possibly improve the absorption and digestibility of other nutrients, such as minerals and vitamins [53].

The addition of biodegradation agents had a significant positive effect on the ND and IBD titers of antibodies. Compared to the control, all levels of supplementation with the biodegradation agent improved the antibody titer but adding 3.75 g kg^−1^ of *S. Cerevisiae* had the best antibody titers against ND and IBD. These obtained results were in accordance with that of Girish and Devegowda [54], who reported that the addition of hydrated sodium calcium aluminosilicate as a biodegrading agent (*p* < 0.05) improved the antibody titers in aflatoxin-fed groups against ND and IBD. Furthermore, improved antibody production in the bursa of Fabricius as an effect of esterified glucomannan supplementation was observed [55]. This result could have been attributed to its binding capacity to mycotoxin and/or its indirect effects on cellular immunity by activating B cells, T cells, and macrophages. Such beneficial effects may be related to the mannan oligosaccharide found in the cell wall of the yeast [55]. The mannan isolated from the *Saccharomyces cerevisiae* cell wall and accompanied by glucan appears to be basically bound with several common mycotoxins [56]. Likewise, the supplementation of T-2 toxins contaminated diets with glucomannan resulted in a significant reduction in lipid peroxidation in the liver of quails [57]. Consequently, the beneficial effects on immune response are primarily related to the mycotoxin binding ability of the biodegradation agent [55].

## 5. Conclusions

Based on this feeding trial results, it can be concluded that the supplementation of a mycotoxin-contaminated diet (100 ppb kg^−1^) with 2.5 or 3.75 g kg^−1^ *Saccharomyces cerevisiae* as a biodegrading agent could enhance broiler performance and feed efficiency as well as the carcass traits. In addition, serum antibodies titers activity against ND and IBD infection diseases were significantly improved. 

## Figures and Tables

**Table 1:** Diet formulation and chemical analysis:

Ingredients	Starter Diet (%)	Finisher Diet (%)
Maize	52.3	54.5
Corn Gluten Meal 30%	2.5	0
Corn Gluten Meal 60%	2.5	1.6
Canola Meal	15	14
Poultry by Product Meal ^1^	4	6
Soybean Meal (Hi-Pro)	19	17.8
Poultry Oil ^2^	2	3.8
Limestone	1	0.9
Salt	0.1	0.1
Di-calcium Phosphate	0.4	0.25
Sodium Bi Carbonate	0.18	0.2
Lysine Sulphate 70%	0.43	0.32
DL-Methionine 99%	0.19	0.19
L-Threonine	0.08	0.02
Premix ^3^	0.32	0.32
Total	100	100
**Chemical composition**		
Crude Protein	23%	22%
Metabolizable Energy	2900 Kcal/Kg	3050 Kcal/Kg
Dig. Lysine ^4^	1.27%	1.19%

^1^ Poultry by-product meal, pet food grade, Griffin Industries Inc., Bastrop, Texas, 66.3% crude protein (CP); ^2^ Poultry oil, Berg and Schmidt India, Hamburg, Germany, 99% crude fat, 8700 Kcal metabolic energy; ^3^ each 1 kg of mineral premix and vitamin contain; Choline Chloride 70%, Betain HCL 98%, Diclazuril 1%, Kemzyme, Vitamin A 20,000,000 I.U., Vit-D_3_ 6,000,000 I.U., Vit-E 60,000 mg, Vit-K3 4000 mg, Vit-B_1_ 4000 mg, Vit-B_2_ 12,000 mg, Vit-B_6_ 8000 mg, Vit-B_12_ 20,000 mg, Nicotinamide 80,000 mg, Biotine 200,000 mg, Folic acid 2000 mg, Ca d-pantothenate 20,000 mg, Manganese 150,000 mg, Zinc 120,000 mg, Iron 96,000 mg, Copper 20,000 mg, Iodine 2000 mg, Selenium 400 mg; ^4^ Dig. Lysine, Digestible Lysine.

**Table 2:** Vaccination schedule program.

Day	Vaccine	Type	Route
1st	IBD (Infectious bursal disease)	Live virus vaccine	Injection Subcutaneous on neck
	IB (Infectious bronchitis—serotype Massachusetts (Strain Ma5)	Live virus vaccine	Eye Drop
	ND (Newcastle disease) virus strain Clone 30	Live virus vaccine	Eye Drop
9th	ND (Strain Ulster 2C (ND)	Killed virus vaccine	Injection Subcutaneous on neck
	H9 (H9N2 Middle East Avian influenza)		
20th	ND	Live attenuated virus vaccine	Drinking water

**Table 3:** Effect of biodegradation agent supplementation on growth performance of broilers during the Hoarder phase (1–35 days) (*n* = 5).

Parameters (g)	Treatments					*p*-Value	
	A	B	C	D	SEM	Linear	Quadratic
BWG (g)	1776 ^d^	1814 ^c^	1944 ^b^	2004 ^a^	9.985	0.00	0.274
FI (g)	3094 ^c^	3087 ^c^	3110 ^b^	3130 ^a^	2.931	0.00	0.00
FCR (%)	1.74 ^a^	1.70 ^b^	1.60 ^c^	1.56 ^d^	0.009	0.00	0.90
Mortality rate (%)	4.0	4.0	4.0	0.0	2.12	0.224	0.36

^a,b,c,d^ Means within a row sharing different superscripts differ significantly (*p* < 0.05). Level of Significance = 0.05; SEM = Standard Error of Mean. A, B, C, D indicate the inclusion of toxin binder at the rate 0.0, 1.25, 2.5, 3.75 g kg^−1^ of *Saccharomyces cerevisiae*, respectively.

**Table 4:** Effect of biodegradation agent supplementation on carcass characteristics of broilers (*n* = 10).

Parameters (g)	Treatments					*p*-Value	
	A	B	C	D	SEM	Linear	Quadratic
Live Body weight	1776 ^d^	1814 ^c^	1944 ^b^	2004 ^a^	2.985	0.00	0.274
Carcass weight	1028 ^d^	1083 ^c^	1196 ^b^	1241 ^a^	1.856	0.000	0.507
Dressing percentage	56.61 ^d^	58.24 ^c^	59.50 ^b^	62.69 ^a^	0.199	0.000	0.001
breast muscle	331.47 ^d^	386.53 ^c^	484.33 ^b^	513.20 ^a^	5.512	0.000	0.030
Relative heart weight	0.63 ^b^	0.64 ^a,b^	0.61 ^a^	0.59 ^a,b^	0.171	0.034	0.224
Relative liver weight	6.21 ^a^	5.18 ^b^	3.53 ^c^	3.04 ^d^	2.512	0.000	0.062
Relative gizzard weight	2.10	2.08	1.92	1.89	0.401	0.476	1.058
Relative proventriculus weight	0.64	0.64	0.60	0.58	0.201	0.217	0.657
Relative kidney weight	0.62	0.61	0.57	0.53	0.345	0.521	0.451
Relative intestine weight	0.71 ^a^	0.64 ^b^	0.55 ^c^	0.04 ^d^	1.335	0.000	0.012
Relative drumsticks weight	19.29 ^d^	23.35 ^c^	26.07 ^b^	27.40 ^a^	7.514	0.000	0.021

^a,b,c,d^ Means within a row sharing different superscripts differ significantly (*p* < 0.05). Level of Significance = 0.05, SEM = Standard Error of Mean. A, B, C, D indicate the inclusion of toxin binder at the rate 0.0, 1.25, 2.5, 3.75 g kg^−1^ of *Saccharomyces cerevisiae*, respectively.

**Table 5:** Mean values of antibody titer against Newcastle disease (ND) and infectious bursal disease (IBD) per bird fed diets containing various levels of Toxin binder (*n* = 10).

Parameters	Treatments					*p*-Value	
	A	B	C	D	SEM	Linear	Quadratic
ND	56 ^d^	79 ^c^	102 ^b^	122 ^a^	1.581	0.00	0.397
IBD	59 ^d^	72 ^c^	81 ^b^	89 ^a^	1.936	0.00	0.266

^a,b,c,d^ Means within a row sharing different superscripts differ significantly (*p* < 0.05). Level of Significance = 0.05, SEM= Standard Error of Mean. a, b, c, d indicate the inclusion of toxin binder at the rate 0.0, 1.25, 2.5, 3.75 g kg^−1^ of *Saccharomyces cerevisiae*, respectively.
